# Age- and environment-dependent changes in chemical defences of larval and post-metamorphic toads

**DOI:** 10.1186/s12862-017-0956-5

**Published:** 2017-06-13

**Authors:** Bálint Üveges, Gábor Fera, Ágnes M. Móricz, Dániel Krüzselyi, Veronika Bókony, Attila Hettyey

**Affiliations:** 1 Lendület Evolutionary Ecology Research Group, Plant Protection Institute, Centre for Agricultural Research, Hungarian Academy of Sciences, Herman Ottó út 15, Budapest, 1022 Hungary; 2 Department of Pathophysiology, Plant Protection Institute, Centre for Agricultural Research, Hungarian Academy of Sciences, Herman Ottó út 15, Budapest, 1022 Hungary

**Keywords:** Bufadienolide, Food limitation, Phenotypic plasticity, Predation risk, Tadpole, Toxin production

## Abstract

**Background:**

Chemical defences are widespread in animals, but how their production is adjusted to ecological conditions is poorly known. Optimal defence theory predicts that inducible defences are favoured over constitutive defences when toxin production is costly and the need for it varies across environments. However, if some environmental changes occur predictably (e.g. coupled to transitions during ontogeny), whereas others are unpredictable (e.g. predation, food availability), changes in defences may have constitutive as well as plastic elements. To investigate this phenomenon, we raised common toad (*Bufo bufo*) tadpoles with ad libitum or limited food and in the presence or absence of chemical cues on predation risk, and measured their toxin content on 5 occasions during early ontogeny.

**Results:**

The number of compounds showed limited variation with age in tadpoles and was unaffected by food limitation and predator cues. The total amount of bufadienolides first increased and later decreased during development, and it was elevated in young and mid-aged tadpoles with limited food availability compared to their ad libitum fed conspecifics, whereas it did not change in response to cues on predation risk. We provide the first evidence for the active synthesis of defensive toxin compounds this early during ontogeny in amphibians. Furthermore, the observation of increased quantities of bufadienolides in food-restricted tadpoles is the first experimental demonstration of resource-dependent induction of elevated de novo toxin production, suggesting a role for bufadienolides in allelopathy.

**Conclusions:**

Our study shows that the evolution of phenotypic plasticity in chemical defences may depend on the ecological context (i.e. predation vs. competition). Our results furthermore suggest that the age-dependent changes in the diversity of toxin compounds in developing toads may be fixed (i.e., constitutive), timed for the developmental stages in which they are most reliant on their chemical arsenal, whereas inducible plasticity may prevail in the amount of synthesized compounds.

**Electronic supplementary material:**

The online version of this article (doi:10.1186/s12862-017-0956-5) contains supplementary material, which is available to authorized users.

## Background

Chemical defences are widespread across the animal kingdom [1, 2] and can serve for deterring predators, parasites, competitors, and pathogens [1–6]. Some species sequester toxic compounds from food or symbionts [4, 6, 7], or obtain them from ambiguous sources [5, 8, 9], while others are capable of de novo synthesizing toxins [3, 4, 10]. However, in species that synthesise toxic compounds themselves, it has remained largely unknown if chemical defences are inducible, i.e. if their production can vary plastically in response to changing environmental conditions [11] and how inducible chemical defences change during ontogeny [12].

Plastic responses are known to evolve under variable environmental conditions and to come with inherent costs [13, 14]. Therefore, induced chemical defences are especially likely to occur in animals that encounter unpredictably varying environments during their lifetime and synthesise toxins de novo, since such synthesis relies on a specialized biochemical pathway and associated physiological and anatomical structures and therefore is considered to be costly [15, 16]. On the other hand, optimal defence theory predicts that changes in chemical defences may become constitutive when environmental differences are predictable [14]; for example, if individuals predictably encounter new environments during their life as a consequence of their development. Consequently, in animals that undergo large, predictable changes in their life-history, and thereby become exposed to drastically different environments that also can unpredictably vary in ecologically important characteristics, chemical defences may both have constitutive as well as inducible components, similarly to other types of defences (e.g. [14, 17, 18]).

Among vertebrates, amphibians undergo the most dramatic changes during their post-embryonic development when they metamorphose and leave the aquatic environment to embark on a terrestrial life [19, 20]. Therefore, amphibians are ideal for studies on ontogenetic changes in toxin production and on the inducibility vs. constitutive nature of chemical defences. Also, chemical defences of vertebrates have been most extensively studied in amphibians. While toxin composition of many amphibian species is well known [3, 7, 21], and experiments documenting age-dependent changes in susceptibility to predators are prevalent in the literature (e.g. [22–26]), in-depth studies on ontogenetic changes in the quantity and composition of toxins utilized in chemical defence and on the underlying secretory apparatus are relatively rare [12, 27–32]. Moreover, there are only a handful of studies on phenotypic plasticity in chemical defences in amphibians [12, 33–35], and only in two of these were larvae sampled for toxin content [12, 35]. Direct evidence for inducible chemical defences in larvae is lacking, and the ability of tadpoles to synthesize toxic compounds has not been confirmed [12]. Also, the studies that so far reported plastic changes in toxin composition in amphibians, and in fact in any vertebrate [12, 33], only documented effects of predators experienced in the larval environment on post-metamorphic animals, while the metamorphic transition from the fully aquatic larval stage to the terrestrial form disrupts selective forces acting during the two life-stages and makes these largely independent of each other. Therefore, evidence for adaptive phenotypic plasticity in chemical defences in species synthesising toxins de novo is lacking.

Here we present a study on the ontogenetic changes and environmental dependence of toxin content in early life-stages of the common toad (*Bufo bufo*). We aimed to (1) examine ontogenetic variation in chemical defences in larval and post-metamorphic common toads and (2) investigate if ontogenetic changes in toxin production may be constitutive or induced by environmental conditions that may affect the pay-off of chemical defence. We experimentally manipulated the presence of chemical cues on predation risk (i.e. the need for toxin production) and food availability (i.e. the costliness of toxin production) and repeatedly assessed the toxin content of individuals during early ontogeny. We predicted that if cues on predation risk are present during tadpole development and tadpoles are able to synthesize toxins themselves, they would start producing such compounds earlier on during their ontogeny and in higher quantities compared to predator-naïve conspecifics. Given that de novo toxin synthesis is considered to be costly [15, 16], we also predicted that food restriction would lead to decreased production of defensive chemicals, manifesting in lowered quantities and decreased numbers of compounds compared to well-fed conspecifics. We chose the common toad as the study species, because it displays relatively weak inducible defences during the larval stage in terms of morphology and behaviour [36–38] and appears to be unpalatable to several predator species [39, 40], suggesting heavy reliance on chemical defence. Also, the chemical composition of *Bufo* skin secretions is relatively well known, their main defensive chemicals being bufadienolides and biogenic amines [41–44], and *B. bufo* are known to contain toxins already in the larval stages [41, 45].

## Methods

### Experimental procedures

In early spring 2013 we collected 10 common toad pairs from a lake in the Pilis Mountains, Hungary (47° 37′ 24.78″ N, 18° 48′ 27.20″ E) and transported them to the experimental station of the Plant Protection Institute (Centre for Agricultural Research, Hungarian Academy of Sciences) in Budapest. We let the pairs spawn in 200-L containers placed outdoors, filled with 60 L of aged tap water and containing twigs as egg-deposition substrates. After egg-laying, we transferred eggs from each clutch to the laboratory, and placed them into dishpans filled with reconstituted soft water (RSW; [46]) to a depth of 2 cm. Temperature was set to 17 °C at the beginning and was allowed to gradually increase to 22 °C by the end of the experiment. We set the lighting to a 13: 11 h light: dark cycle.

Upon hatching, we haphazardly selected four hatchlings of each family and stored them in 70% HPLC-grade methanol, resulting in 40 samples collected at the start of the experiment. Hatchlings were at developmental stage 19 ([20], Additional file 1: Figure S1). We used this sampling to estimate the baseline of bufadienolide content at the start of larval development. We further assigned randomly selected hatchlings in groups of three to 2-L containers filled with 1.5 L RSW, distributed randomly among treatments. We employed a three-factorial design with two predator-cue treatments (control vs. chemical cues on predation risk), two food level treatments (ad libitum vs. limited food), and four sampling occasions during the larval and early metamorphic life-stages (for details see below). We replicated each combination of predator treatment × food level treatment × sampling occasion 20 times, resulting in a total of 320 experimental units at the start of the experiment. Each family was represented twice in each treatment combination. Containers were arranged in groups of four in a randomized block design, where each block contained tadpoles from one family.

As predators we used five 4th instar larvae of the southern hawker, *Aeshna cyanea*, and five adult, male smooth newts, *Lissotriton vulgaris*. We kept individuals of both species grouped in 5-L containers filled with 3 L of RSW, and fed them daily with 800 mg *B. bufo* and 800 mg agile frog, *Rana dalmatina*, tadpoles each. We prepared stimulus water by mixing the water taken from the tanks in which we housed and fed the predators, and simulated predation risk by transferring 30 ml stimulus water daily to the rearing containers of tadpoles in the predator-cue treatment group, while adding equal amounts of RSW to the containers of the control group.

We fed tadpoles with a finely ground 4:1 mixture of rabbit chow and fish flakes. Tadpoles assigned to the ad libitum food treatment group received a food amount of ca. 12% of their body mass/individual/day; tadpoles in the limited food treatment group received one-third of that amount. We adjusted food quantity by weighing tadpoles to the nearest mg at the sampling occasions (see below, Additional file 1: Figure S2). We changed the water in the tadpoles’ rearing containers every third day. Whenever we observed a dead individual, we removed it, but spontaneous mortality remained relatively low during the experiment (110 tadpoles out of 960, 11.46%).

When tadpoles were approaching metamorphosis, we monitored the rearing containers twice a day. When the first tadpole in a container started to metamorphose (appearance of at least one forelimb, developmental stage 42 according to [20]), we removed the other individuals from that container, decreased the water level to 1 dl and slightly tilted the containers to allow the metamorph to leave the water.

After the sampling of hatchlings at the start of the experiment, we took samples for toxin analysis four more times, preserving 40 individuals at each occasion [47]. The second and third samplings took place after 14 and 21 days, when tadpoles reached the median developmental stages of 28 (range = 28–30) and 34 (31–36), respectively. We took a fourth sample when tadpoles reached a median developmental stage of 38 (37–41). The date of this sampling occasion was not specified a priori, but was rather determined based on how developed tadpoles were (the presence of well-formed hind limbs), to account for potential differences in growth rate between treatment groups. We performed a final sampling when individuals completed metamorphosis (complete disappearance of the tail at stage 46, Additional file 1: Figure S1). Each container was sampled once during the entire experiment, by haphazardly selecting and conserving one individual from it. From the 320 experimental containers we therefore collected 320 samples, half of which we later analysed for toxin content. In each treatment × family combination, one container was a priori designated to chemical analysis while the other container was used as a backup; the latter samples were analysed only if we encountered problems during sample preparation for HPLC of the respective a priori sample (21 instances out of 160 samples, 13.13%, [47]). We released adults, unused eggs and all remaining tadpoles, metamorphs and toadlets at the site of collection.

### Analysis of toxin content

**Fig. 1:**
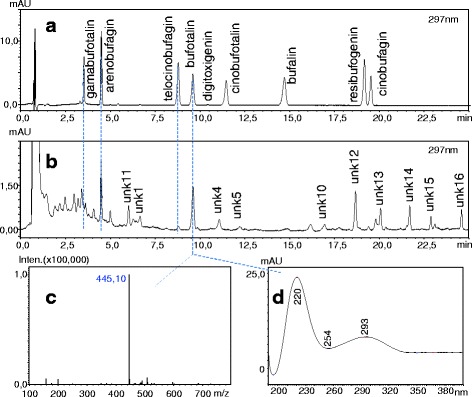
HPLC-DAD-ESI-MS analysis of bufadienolides. Representative UV chromatograms of the separated standards (**a**) and a common toad sample (**b**, sample nr. 213 [47]), as well as the MS (**c**) and UV (**d**) spectra of bufotalin. Further examples of representative chromatograms of the common toad can be accessed in the online appendix of [45]

We used high-performance liquid chromatography with diode-array detection and mass spectrometry (HPLC-DAD-MS) to identify and quantify bufadienolide compounds. We homogenized specimens using a homogenizer (VWR VDI 12) with a dispersing tool (IKA S12 N-7S). After drying samples in vacuo at 45 °C using a rotary evaporator (Büchi Rotavapor R-134), we measured dry weight of samples using an analytical balance (Ohaus Pioneer PA-114) to the nearest 0.1 mg and subsequently re-dissolved samples in 1 ml HPLC-grade absolute methanol, which was further aided by exposing the samples briefly to ultrasound in a bath sonicator (Tesla UC005AJ1). We filtered the samples using nylon syringe filters (FilterBio, pore size = 0.22 μm). We identified compounds as bufadienolides by inspecting the UV spectrum of peaks [27, 33, 45] and by using commercially acquired bufalin, bufotalin, resibufogenin, gamabufotalin, areno- and telocinobufagin (Biopurify Phytochemicals, Chengdu, China), cinobufagin (Chembest, Shanghai, China), cinobufotalin (Quality Phytochemicals, New Jersey, USA) and digitoxigenin (Santa Cruz Biotechnology, Dallas, TX, USA) as standards (Fig. 1). Identification of compounds present in low quantities was further aided by the analysis of a sample obtained from an adult male common toad by gently massaging the parotoid glands.

A single-quadrupole HPLC-MS system (Shimadzu LC-MS-2020) equipped with a binary gradient solvent pump, a vacuum degasser, a thermostated autosampler, a column oven, a diode array detector and a mass analyser with electrospray ionization (ESI-MS) was used. Chromatographic separations were carried out at 35 °C on a C18 2.6 μm column (Kinetex, 100 mm × 3 mm i.d.) in series with a C18 guard column (4 mm × 3 mm i.d.) using 10 μL injections. The mobile phase consisted of water containing 0.05% formic acid (solvent A) and acetonitrile containing 0.05% formic acid (solvent B). The flow rate was 0.8 mL/min and the gradient was as follows: 0–2 min, 15–25% B; 2–15 min, 25–35% B; 15–24 min, 30–50% B; 24–25 min, 50–90% B; 25–30 min 90% B; 30–35 min 15% B. ESI worked under the following conditions: desolvation line (DL) temperature, 250 °C; heat block temperature, 400 °C; drying N_2_ gas flow, 15 L/min; nebulizer N_2_ gas flow, 1.5 L/min; positive ionization mode. Data was acquired and processed using the programme LabSolutions 5.42v (Shimadzu).

### Statistical analyses

To calculate the number of bufadienolide compounds (NBC) present in each animal, we assumed a compound to be present when its area value was larger than zero in the chromatogram (Fig. 1). We estimated the quantity of each compound from the area values of chromatogram peaks (Fig. 1) based on the calibration curve of the bufotalin standard, and summed up these values to obtain an estimate of total bufadienolide quantity (TBQ) for each individual. We used the calibration curve of the bufotalin standard, because this was the most ubiquitously identified compound in our samples (Table 1). This approach yields approximate estimates of bufadienolide quantities, but it has been successfully used before in similar studies [12, 33, 45].

**Table 1:** Percent occurrence, retention time and mass signal of bufadienolides in common toad tadpoles

Compound name	Percent occurrence of bufadienolide compounds					retention time (min)	m/z [M + H]^+^
	stage 19	stage 28	stage 34	stage 38	stage 46		
Arenobufagin	10	67.5	90	95	43.6	4.5	417.2
Bufalin	-	25	40	60	56.4	14.5	387.25
Bufotalin	5	97.5	95	97.5	100	9.5	445.3
Gamabufotalin	-	-	15	35	51.3	3.5	403.25
Resibufogenin	-	12.5	5	17.5	15.4	19	385.25
Telocinobufagin	22.5	37.5	47.5	70	100	8.7	403.25
unidentified bufadienolide 1	-	100	100	100	100	6.6	729.35
unidentified bufadienolide 2	2.5	95	92.5	95	100	7.5	727.3
unidentified bufadienolide 3	-	5	-	2.5	51.3	9.6	729
unidentified bufadienolide 4	-	92.5	100	100	100	10.6	715
unidentified bufadienolide 5	-	10	12.5	5	-	11.8	627.4
unidentified bufadienolide 6	-	72.5	72.5	60	94.9	12.3	713.3
unidentified bufadienolide 7	-	12.5	25	2.5	53.8	12.9	671.35
unidentified bufadienolide 8	-	85	90	85	76.9	13	743
unidentified bufadienolide 9	-	75	85	85	-	16.7	671.4
unidentified bufadienolide 10	-	100	85	82.5	97.4	16.9	757.3
unidentified bufadienolide 11	7.5	40	75	87.5	66.7	6	415.3
unidentified bufadienolide 12	2.5	100	97.5	100	56.4	18.6	573.15
unidentified bufadienolide 13	2.5	100	100	100	28.2	20	571.1
unidentified bufadienolide 14	-	100	100	95	82.1	21.7	367.1
unidentified bufadienolide 15	2.5	85	25	10	15.4	22.9	365.1
unidentified bufadienolide 16	-	100	100	97.5	84.6	24.6	601.15

Compounds represented by "-" were not detectable by HPLC-DAD-MS in any of the samples

**Fig. 2:**
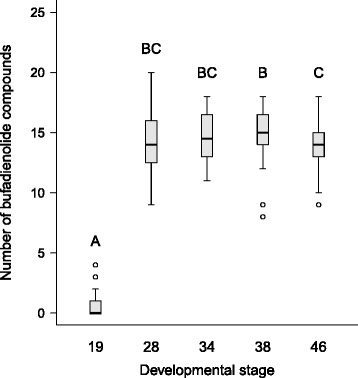
Number of bufadienolide compounds of common toad hatchlings, tadpoles and post-metamorphs during ontogeny (*N* = 199). Letters above boxplots indicate homogeneous subsets according to pairwise comparisons corrected for false discovery rate. In each boxplot, the thick horizontal line and the box represent the median and the interquartile range, respectively; whiskers extend to the upper and lower quartile ±1.5 × interquartile range; open circles represent outliers. Statistics for pairwise comparisons can be found in Additional file 1: Table S2

**Fig. 3:**
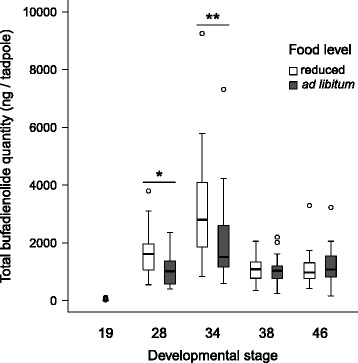
Total bufadienolide quantity of common toad hatchlings, tadpoles and post-metamorphs (*N* = 198). Asterisks above boxplots represent pairwise comparisons between food level treatments; groups marked with * (*P* < 0.05) and ** (*P* = 0.01) differ significantly based on linear contrasts corrected for false discovery rate. Food level treatment was applied after developmental stage 19 (see Methods). Statistics for pairwise comparisons can be found in Additional file 1: Tables S2 and S3

We analysed the effects of predator-cue, food treatments and developmental stage on toxin content using linear mixed-effects models (LMM). We entered NBC or TBQ as the dependent variable; we used the log_10_-transformed values of TBQ to ensure normality of model residuals and homogeneity of variances. Initial models included food level, predator-cue treatment, and developmental stage of tadpoles as fixed factors, their two-way and three-way interactions; and block nested within family as random factors. In the analyses of TBQ, we also entered the log_10_-transformed dry mass of tadpoles as a covariate, but without interactions with the other explanatory variables. Note that the data obtained from the first sampling occasion (developmental stage 19) were not included in the LMM analyses, because treatments were only applied after this stage. With each initial model, we performed a backward model-simplification procedure based on *P-*values, with α = 0.05. To calculate relevant statistics for non-significant terms that were dropped during model selection, we re-entered the removed variables one by one into the final models. We ran all analyses in R 3.1.3 [48] using the ‘lme’ function in the ‘nlme’ package [49]. *P-*values were calculated using ‘anova’ in ‘nlme’, using type-3 sums of squares. We conducted pairwise comparisons among treatment groups and samplings by calculating linear contrasts corrected for false discovery rate [50] using the ‘lsmeans’ package [51]. Model residuals of NBC and TBQ showed considerable heteroscedasticity between samplings when developmental stage 19 was included in the analysis (Figs. 2 and 3), therefore in these instances we allowed for different within-sampling variances using ‘weights’ with ‘varIdent’ in ‘nlme’ [49]. We also analysed the quantity of each bufadienolide compound separately; the final models of these analyses are presented in the supplementary information (Additional file 1: Table S1). We had to discard one sample from the analysis on NBC, and two samples from the analysis on TBQ due to missing data [47].

## Results

### Number of bufadienolide compounds

Toad hatchlings (developmental stage 19) contained a small number of bufadienolides or none at all (median: 0, range: 0-4, *N* = 40; 25 hatchlings (62.5%) did not contain any compounds in detectable quantities). In contrast, bufadienolides had high diversity in all other age categories (developmental stages 28–46, median NBC: 14, range: 8–20, *N* = 159; Table 1, Fig. 2). One compound (unidentified bufadienolide 1) was found in all tadpoles and post-metamorphic individuals (Table 1). After hatching, the effect of developmental stage on NBC was marginally non-significant (Table 2), as tadpoles in any stage did not differ from each other significantly, while there was a small but significant difference between post-metamorphic toads and metamorphosing individuals such that the post-metamorphs had slightly fewer (ca. 1 compound less) bufadienolides (Fig. 2, Additional file 1: Table S2). Predation risk and food limitation did not have a significant effect on NBC (Table 2, Additional file 1: Figure S3).

**Table 2:** Effects of ontogeny, treatments, their interactions, and body mass on bufadienolide synthesis of common toads

	*N*	df	F	*P*
*Number of bufadienolide compounds (NBC)*	159			
**intercept**		**1, 80**	**860.495**	**<0.0001**
developmental stage		3, 80	2.222	0.092
food level		1, 82	0.018	0.894
predation treatment		1, 82	0.442	0.508
developmental stage × food level		3, 76	1.882	0.140
developmental stage × predation treatment		3, 76	0.249	0.862
food level × predation treatment		1, 80	0.266	0.608
developmental stage × food level × predation treatment		3, 68	0.368	0.777
*Total bufadienolide quantity (TBQ, ng)*	158			
**intercept**		**1, 78**	**3726.423**	**<0.0001**
body mass		1, 77	2.342	0.130
**developmental stage**		**3, 78**	**18.313**	**<0.0001**
**food level**		**1, 78**	**9.646**	**0.003**
predation treatment		1, 77	0.495	0.484
developmental stage × food level		3, 75	2.360	0.078
developmental stage × predation treatment		3, 74	0.358	0.784
food level × predation treatment		1, 76	0.493	0.485
developmental stage × food level × predation treatment		3, 67	0.754	0.524

Terms present in the final models are highlighted in bold. Statistics for non-significant terms that were dropped during model selection were calculated by re-entering the removed variables one by one into the final models

### Total bufadienolide quantity

Toad hatchlings contained only minute amounts of bufadienolides (mean ± SE: 12.603 ± 4.065 ng / tadpole, *N* = 40) compared to all other age categories (mean ± SE: 1555.864 ± 97.796 ng / tadpole, *N* = 158, Fig. 3). The compound that was present in all individuals after the hatchling stage had the highest mean quantity (unidentified bufadienolide 1; mean ± SE: 313.738 ± 30.424 ng/tadpole). Across the tadpole stages, the total quantity of bufadienolides increased significantly to developmental stage 34 but decreased afterwards (Table 2, Fig. 3, Additional file 1: Table S2). Furthermore, during the first half of tadpole development (developmental stages 28 and 34), tadpoles that received reduced amounts of food contained significantly more bufadienolides than their ad libitum fed conspecifics (Table 2, Fig. 3, Additional file 1: Table S3), despite having significantly lower body mass (LMM of body mass, age: F_3, 214_ = 160.694, *P* = <0.0001; food level: F_1, 214_ = 26.831, *P* = <0.0001; age × food level: F_3, 214_ = 34.735, *P* = <0.0001; *N* = 303; Additional file 1: Figure S2), whereas this difference in TBQ disappeared in more developed tadpoles and post-metamorphic individuals (Fig. 3, Additional file 1: Table S3). Presence or absence of chemical cues on predation risk did not influence TBQ (Table 2, Additional file 1: Figure S4). Analysing the quantity of each bufadienolide compound separately corroborated our findings that toxin content varied with age and food level but not with predation-cue treatment (Additional file 1: Table S1).

## Discussion

Our study is the first to unequivocally demonstrate de novo production of toxic compounds in amphibian larvae, as indicated by the steep increase in both the number and quantity of bufadienolide compounds after hatching. This finding shows that common toad tadpoles synthesize their toxins de novo, as has been suggested by histological and ultrastructural studies that demonstrated the presence of the underlying secretory cells and glands already during larval life [30, 31]. This contrasts with other toad species [12, 27] in which tadpoles were found not to produce bufadienolides, relying instead on maternal provisioning of these toxins. For example, in the cane toad (*Rhinella marina*) [27] the diversity and amount of bufadienolides are highest in eggs and gradually decrease until developmental stage 25 [20]. Because we were primarily interested in the phenotypic plasticity of toxin production, we did not investigate eggs, so it remains possible that the same decrease from the egg stage to hatching occurs in common toads. This is supported by observations that common toad eggs are repulsive to many different predator species [39, 40], although compounds other than bufadienolides (e.g. biogenic amines) may also be responsible for the unpalatability of common toad eggs (and hatchlings), as suggested for larvae of *A. boreas* [12]. Nonetheless, because the majority of hatchlings in our study contained no bufadienolides at all, the importance of maternal provisioning of these toxins appears to be limited in common toads. Clearly, maternal toxin provisioning and the temporal changes in toxin content during embryonic development of common toads demands further investigation.

We found that bufadienolides accumulated quickly in young tadpoles and, after reaching a peak in mid-aged larvae, decreased to lower quantities as metamorphosis was approaching. This pattern mirrors ontogenetic changes in tadpole vulnerability: young tadpoles are more vulnerable to predators, thus early toxin production may be strongly favoured. Later, when tadpoles grow larger, they reach a size refuge against several predators or are more difficult to capture [23–26], therefore they may have to rely less on chemical defences. Such an adjustment of toxin dosage to vulnerability to predators may be common in chemically defended organisms [52–54]. Additionally, the ability of predators to distinguish and learn to avoid noxious prey [55–57] may relieve tadpoles from synthesising large amounts of toxins in later stages. Despite a decrease in total bufadienolide quantity, metamorphosing and post-metamorphic individuals in our study still contained considerable amounts of bufadienolides, most likely providing them with effective defences against certain predators and perhaps also pathogens and parasites [58, 59], although metamorphosing anurans (between developmental stages 42 and 46) are more susceptible to predation than late tadpole stages or already metamorphosed animals [60–64]. The decreased bufadienolide quantity we observed in these later stages may be attributed to proximate constraints associated with metamorphosis, when a complete re-organization of several physiological systems occurs [19, 65, 66]. We would expect a similar pattern also in other animal species that actively synthesise toxins and undergo substantial morphological and physiological changes during their ontogeny, such as many insects [67].

The observation that predation risk did not induce the production of larger quantities of bufadienolides in tadpoles is surprising, although it agrees with our earlier finding that, in natural ponds, the toxin content of toad tadpoles did not correlate with the density of predators [45]. A previous experimental study did not observe inducible changes in chemical defences of tadpoles either, but this was attributable to the lack of toxin synthesis in tadpoles of the study species [12]. One possible explanation in our case is that predator-induced changes in chemical defence exist in common toad tadpoles, but not in response to the specific predators we used. However, previous studies did document plastic changes in life-history traits, behaviour and morphology of common toad tadpoles to chemical cues on the presence of *A. cyanea* [38, 68], suggesting that they can detect their presence based on olfactory cues, perceive these predators as dangerous, and respond to them by changes in life-history traits. Because fishes are considered to be the most voracious predators of amphibian larvae [69], it is possible that in certain populations that live in permanent ponds, such as the one used in the current study, a relatively high baseline level of bufadienolide synthesis becomes fixed via selection [70]. Alternatively, predator-induced plasticity of toxin production may be lacking in toad tadpoles in general, perhaps because of the toxins’ apparent low production cost [71], and because very high spatiotemporal variability of predator communities may favour constitutive defences [45]. Finally, although bufadienolides may be effective in repelling several predators, it is possible that the evolution of plasticity in toxin production is driven by other factors, such as pathogens [72, 73] or competitors ([45], see below).

Our results demonstrated inducible changes in toxin production in response to food availability: during early larval life, food-deprived tadpoles contained significantly more bufadienolides than their ad libitum-fed conspecifics. This result, combined with the fact that toxin content was not related to body mass, corroborates our earlier finding that the energetic costs of toxin production in toad larvae may be low [71]. It seems contradictory that an inducible defence may be cheap to produce, but detecting associated costs of expressed plastic traits may be problematic in species with a complex life-history, such as anurans, because costs may not appear synchronously with the displayed trait [74–77]. Nonetheless, enhanced toxin production in food-limited tadpoles concords with results of our field study showing that common toad tadpoles in ponds with high density of competitors (mainly amphibian larvae) contained more bufadienolide compounds and slightly larger total quantities of bufadienolides than tadpoles coexisting with fewer competitors [45]. Thus, in the current experiment, reduced food level might have acted as an indicator of high competitor density, inducing the synthesis of larger amounts of bufadienolides against competitors or the pathogens and parasites they carry. Allelopathy, which is intra- or interspecific competition mediated by chemical substances [78], is a phenomenon of fundamental importance in algae and plants [79, 80], but for animals it has been rarely reported so far [81–83]. The existence of chemical interference between amphibian larvae was proposed long ago, but the mediating agents involved in the process have not been identified [69, 84]. Bufadienolides have been suggested to act as allelochemicals [83], but it remains to be tested directly whether the synthesis of these compounds benefits toad tadpoles by negatively affecting competitors or naturally occurring pathogens and parasites. Nonetheless, our results suggest that allelopathy may be a significant factor in the ecology of a wider variety of animals than currently thought.

## Conclusions

In conclusion, our results are the first to document plastic changes in chemical defences in response to food availability in any vertebrate capable of de novo toxin synthesis. The observation that tadpoles produced more toxins at low food availability than when food was present ad libitum indicated that bufadienolides may be relatively cheap to produce but their production may respond plastically to the perceived intensity of competition for food. Our results furthermore suggest that the ontogenetic timing of the production of various toxin components may be fixed as a constitutive defence in toad tadpoles, whereas inducible plasticity prevails in how much of these components is produced. These results, coupled with those of previous studies, highlight the existence of surprisingly diverse strategies of toxin provisioning and synthesis even among as closely related taxa as the species of the Bufonidae family and, thus, caution against premature generalization of observed strategies among species of other chemically defended groups of organisms. Our findings also suggest that ontogenetic changes in toxin production may have resulted from adaptation to predictable variation in predation risk over development, and, thus, represent constitutive age-dependent changes in anti-predator defence rather than a phenotypically plastic response. Therefore, the same trait can show different degrees of phenotypic plasticity depending on evolutionary history (i.e. different species) and ecological context (e.g. predators or other enemies, such as competitors). Studies scrutinizing the costs of toxin production, clarifying the role of toxins in competitive interactions and immune defence, and identifying the environmental factors promoting fixation of the rate of toxin synthesis appear to be especially promising avenues of future research and will provide important insights into the evolution and ecology of chemical defences.

## Additional file

Additional file 1: Supplementary methods. Supplementary results. **Tables S1–S4.** **Figures S1–S4.** (DOCX 238 kb)
