# Supplementary material for: Age- and environment-dependent changes in chemical defences of larval and post-metamorphic toads
Source: BMC Evol Biol. 2017 Jun 13;17:137. doi: 10.1186/s12862-017-0956-5 (PMC5470210; doi:10.1186/s12862-017-0956-5)
Supplement: Additional file 1: — Supplementary methods. Supplementary results. Tables S1–S4. Figures S1–S4. (DOCX 238 kb) [file 12862_2017_956_MOESM1_ESM.docx]

Supplementary material for

Age- and environment-dependent changes in chemical defences of larval and post-metamorphic toads

Published in BMC Evolutionary Biology

Bálint Üveges^1*^, Gábor Fera^1^, Ágnes M. Móricz^2^, Dániel Krüzselyi^2^, Veronika Bókony^1^, Attila Hettyey^1^

*^1^Lendület Evolutionary Ecology Research Group, Plant Protection Institute, Centre for Agricultural Research, Hungarian Academy of Sciences, Herman Ottóút 15, Budapest, 1022, Hungary*

*^2^Department of Pathophysiology, Plant Protection Institute, Centre for Agricultural Research, Hungarian Academy of Sciences, Herman Ottóút 15, Budapest, 1022, Hungary*

^*^Corresponding author. E-mail:uveges.balint@agrar.mta.hu

**Supplementary methods**

*Statistical analysis of quantity of individual bufadienolide compounds*. We considered to analyse variation in toxin diversity using the method proposed by Marion *et al.* (2015), but our experimental design (three fixed effects with multiple levels) rendered such an approach unfeasible. Therefore, to check if our treatments influenced toxin composition, we settled on analysing each bufadienolide’s quantity individually, using the analytical procedure described in the main text. Not all compounds could be analysed this way, since in many cases initial models did not meet the assumptions of linearity and/or homoscedasticity. This was especially apparent in the case of rare compounds (13 compounds wereabsent in more than 25% of tadpoles). Model residuals of some bufadienolides showed considerable heteroscedasticity between treatment groups; in these instances we allowed for different within-group variances using 'weights' with 'varIdent' in 'nlme' (Pinheiro *et al.* 2015). To improve model fit, some compounds’ quantities were transformed before analysisas log_10_*X* (if the compound was present in all animals) or log_10_(*X*+1) (if the compound was not detected in some animals). Evaluation of model fit was based on visual observation of diagnostic plots. Statistics of the analysed compounds can be found in Supplementary Table 1.

**Supplementary results**

*Wet body mass of* tadpoles. We weighed toad tadpoles to the nearest mg at the sampling occasionsright before conserving some of them in methanol. When fed *ad libitum*, tadpoles that received predatory cues had smaller body mass compared to their predator-naïve conspecifics (LMM of body mass, food level × predator cue: F_1,214_ = 7.764, *P* = 0.006, *N* = 303, Supplementary Table 4, Supplementary Fig.2). This effect could not be observed in tadpoles that received a reduced amount of food (Supplementary Table 4, Supplementary Fig.2), probably because the amount of food was so limited that tadpoles that received predator cues could not afford to reduce their foraging time without risking starvation, and/or because the full amount of food provided could be ingested even with a reduced activity level.

**Supplementary Table 1:** Effects of developmental stage, food level, their interactions, and body mass on the quantity of individual bufadienolide compounds in common toad tadpoles. For brevity, only statistics of the final models are presented. Significant (*P*<0.05) terms are marked with an asterisk ("†" marks a marginally non-significant interaction obtained by adding it to the final model). Statistics of non-significant terms are available from the authors upon request.

| Bufadienolide | *N* |  | df |  | F |  | *P* |
| --- | --- | --- | --- | --- | --- | --- | --- |
|  |  |  |  |  |  |  |  |
|  | 158 |  |  |  |  |  |  |
|  |  |  |  |  |  |  |  |
| *Bufotalin* |  |  |  |  |  |  |  |
| intercept * |  |  | 1, 75 |  | 93.891 |  | <0.0001 |
| developmental stage * |  |  | 3, 75 |  | 19.955 |  | <0.0001 |
| food level * |  |  | 1, 75 |  | 10.052 |  | 0.0022 |
| developmental stage × food level * |  |  | 3, 75 |  | 8.197 |  | 0.0001 |
|  |  |  |  |  |  |  |  |
| *Unidentified bufadienolide 1* |  |  |  |  |  |  |  |
| intercept * |  |  | 1, 78 |  | 604.166 |  | <0.0001 |
| developmental stage * |  |  | 3, 78 |  | 24.537 |  | <0.0001 |
| food level * |  |  | 1, 78 |  | 22.425 |  | <0.0001 |
|  |  |  |  |  |  |  |  |
| *Unidentified bufadienolide 2* |  |  |  |  |  |  |  |
| intercept * |  |  | 1, 75 |  | 171.314 |  | <0.0001 |
| developmental stage * |  |  | 3, 75 |  | 8.421 |  | 0.0001 |
| food level * |  |  | 1, 75 |  | 10.352 |  | 0.0019 |
| developmental stage × food level * |  |  | 3, 75 |  | 4.585 |  | 0.0053 |
|  |  |  |  |  |  |  |  |
| *Unidentified bufadienolide 4* |  |  |  |  |  |  |  |
| intercept * |  |  | 1, 75 |  | 323.246 |  | <0.0001 |
| developmental stage * |  |  | 3, 75 |  | 8.105 |  | 0.0001 |
| food level * |  |  | 1, 75 |  | 12.397 |  | 0.0007 |
| developmental stage × food level * |  |  | 3, 75 |  | 3.771 |  | 0.0141 |
|  |  |  |  |  |  |  |  |
| *Unidentified bufadienolide 6* |  |  |  |  |  |  |  |
| intercept * |  |  | 1, 75 |  | 49.137 |  | <0.0001 |
| developmental stage * |  |  | 3, 75 |  | 11.778 |  | <0.0001 |
| food level |  |  | 1, 75 |  | 2.689 |  | 0.1053 |
| developmental stage × food level * |  |  | 3, 75 |  | 13.995 |  | <0.0001 |
|  |  |  |  |  |  |  |  |
| *Unidentified bufadienolide 8* |  |  |  |  |  |  |  |
| intercept * |  |  | 1, 78 |  | 83.388 |  | <0.0001 |
| developmental stage * |  |  | 3, 78 |  | 6.281 |  | 0.0007 |
| food level * |  |  | 1, 78 |  | 13.359 |  | 0.0005 |
|  |  |  |  |  |  |  |  |
| *Unidentified bufadienolide 10* |  |  |  |  |  |  |  |
| intercept * |  |  | 1, 75 |  | 293.146 |  | <0.0001 |
| developmental stage * |  |  | 3, 75 |  | 13.944 |  | <0.0001 |
| food level |  |  | 1, 75 |  | 2.230 |  | 0.1395 |
| developmental stage × food level * |  |  | 3, 75 |  | 12.236 |  | <0.0001 |
|  |  |  |  |  |  |  |  |
| *Unidentified bufadienolide 14* |  |  |  |  |  |  |  |
| intercept * |  |  | 1, 78 |  | 88.178 |  | <0.0001 |
| body mass * |  |  | 1, 78 |  | 31.279 |  | <0.0001 |
| developmental stage * |  |  | 3, 78 |  | 94.141 |  | <0.0001 |
| food level |  |  | 1, 74 |  | 0.241 |  | 0.625 |
| developmental stage × food level † |  |  | 3, 74 |  | 2.339 |  | 0.0804 |
|  |  |  |  |  |  |  |  |
| *Unidentified bufadienolide 16* |  |  |  |  |  |  |  |
| intercept * |  |  | 1, 78 |  | 104.562 |  | <0.0001 |
| body mass * |  |  | 1, 78 |  | 15.298 |  | 0.0002 |
| developmental stage * |  |  | 3, 78 |  | 186.924 |  | <0.0001 |
|  |  |  |  |  |  |  |  |

**Supplementary Table 2:** Pairwise comparisons of the number and quantity (ng / tadpole) of toxin compounds between different developmental stages of common toads. Total bufadienolide quantity was log_10_(*X*+1) transformed before analysis. Significant differences (FDR-corrected *P* < 0.05) are marked with an asterisk.

| Comparison |  | Difference |  | SE |  | df |  | *t* |  | *P* |
| --- | --- | --- | --- | --- | --- | --- | --- | --- | --- | --- |
|  |  |  |  |  |  |  |  |  |  |  |
| *Number of bufadienolide compounds* |  |  |  |  |  |  |  |  |  |  |
| Developmental stage 19 & 28* |  | -13.575 |  | 0.393 |  | 185 |  | -34.557 |  | <0.0001 |
| Developmental stage 19 & 34* |  | -13.975 |  | 0.353 |  | 185 |  | -39.625 |  | <0.0001 |
| Developmental stage 19 & 38* |  | -14.275 |  | 0.347 |  | 185 |  | -41.193 |  | <0.0001 |
| Developmental stage 19 & 46* |  | -13.170 |  | 0.368 |  | 185 |  | -35.808 |  | <0.0001 |
| Developmental stage 28 & 34 |  | -0.400 |  | 0.492 |  | 185 |  | -0.812 |  | 0.469 |
| Developmental stage 28 & 38 |  | -0.700 |  | 0.488 |  | 185 |  | -1.435 |  | 0.219 |
| Developmental stage 28 & 46 |  | 0.405 |  | 0.503 |  | 185 |  | 0.805 |  | 0.469 |
| Developmental stage 34 & 38 |  | -0.300 |  | 0.456 |  | 185 |  | -0.658 |  | 0.512 |
| Developmental stage 34 & 46 |  | 0.805 |  | 0.473 |  | 185 |  | 1.704 |  | 0.150 |
| Developmental stage 38 & 46* |  | 1.105 |  | 0.468 |  | 185 |  | 2.361 |  | 0.039 |
|  |  |  |  |  |  |  |  |  |  |  |
| *Total bufadienolide quantity* |  |  |  |  |  |  |  |  |  |  |
| Developmental stage 19 & 28* |  | -2.582 |  | 0.114 |  | 184 |  | -22.595 |  | <0.0001 |
| Developmental stage 19 & 34* |  | -2.839 |  | 0.116 |  | 184 |  | -24.398 |  | <0.0001 |
| Developmental stage 19 & 38* |  | -2.501 |  | 0.112 |  | 184 |  | -22.342 |  | <0.0001 |
| Developmental stage 19 & 46* |  | -2.495 |  | 0.117 |  | 184 |  | -21.284 |  | <0.0001 |
| Developmental stage 28 & 34* |  | -0.258 |  | 0.056 |  | 184 |  | -4.569 |  | <0.0001 |
| Developmental stage 28 & 38 |  | 0.081 |  | 0.047 |  | 184 |  | 1.738 |  | 0.105 |
| Developmental stage 28 & 46 |  | 0.087 |  | 0.058 |  | 184 |  | 1.488 |  | 0.154 |
| Developmental stage 34 & 38* |  | 0.339 |  | 0.052 |  | 184 |  | 6.572 |  | <0.0001 |
| Developmental stage 34 & 46* |  | 0.344 |  | 0.062 |  | 184 |  | 5.533 |  | <0.0001 |
| Developmental stage 38 & 46 |  | 0.006 |  | 0.053 |  | 184 |  | 0.105 |  | 0.916 |

**Supplementary Table 3:** Pairwise comparisons of total bufadienolide quantity (ng / tadpole) between food levels within developmental stages of common toads. Total bufadienolide quantity was log_10_*-*transformed before analysis. Significant terms (FDR-corrected *P* < 0.05) are marked with an asterisk.

| Comparison |  | Difference |  | SE |  | df |  | *t* |  | *P* |
| --- | --- | --- | --- | --- | --- | --- | --- | --- | --- | --- |
| Developmental stage 28, reduced & *ad libitum* food* |  | 0.197 |  | 0.074 |  | 75 |  | 2.657 |  | 0.019 |
| Developmental stage 34, reduced & *ad libitum* food* |  | 0.232 |  | 0.074 |  | 75 |  | 3.135 |  | 0.010 |
| Developmental stage 38, reduced & *ad libitum* food |  | 0.017 |  | 0.075 |  | 75 |  | 0.224 |  | 0.824 |
| Developmental stage 46, reduced & *ad libitum* food |  | 0.019 |  | 0.075 |  | 75 |  | 0.247 |  | 0.824 |

**Supplementary Table 4:** Pairwise comparisons of wet body mass (mg) of common toad tadpoles between predator cue treatments within developmental stages and food levels. Significant terms (FDR-corrected P < 0.05) are marked with an asterisk ("†" depicts a marginally non-significant difference).

| Comparison |  | Difference |  | SE |  | df |  | *t* |  | *P* |
| --- | --- | --- | --- | --- | --- | --- | --- | --- | --- | --- |
| Developmental stage 28, reduced food, control & predator cues |  | 0.750 |  | 9.388 |  | 208 |  | 0.080 |  | 0.936 |
| Developmental stage 34, reduced food, control & predator cues |  | 4.635 |  | 9.646 |  | 208 |  | 0.481 |  | 0.631 |
| Developmental stage 38, reduced food, control & predator cues |  | -1.943 |  | 9.646 |  | 208 |  | -0.201 |  | 0.841 |
| Developmental stage 46, reduced food, control & predator cues |  | -4.791 |  | 9.766 |  | 208 |  | -0.491 |  | 0.624 |
| Developmental stage 28, *ad libitum* food, control & predator cues |  | -2.500 |  | 9.388 |  | 208 |  | -0.266 |  | 0.790 |
| Developmental stage 34, *ad libitum* food, control & predator cues * |  | 38.284 |  | 9.511 |  | 208 |  | 4.025 |  | 0.0001 |
| Developmental stage 38, *ad libitum* food, control & predator cues † |  | 17.968 |  | 9.765 |  | 208 |  | 1.840 |  | 0.067 |
| Developmental stage 46, *ad libitum* food, control & predator cues * |  | 22.883 |  | 10.255 |  | 208 |  | 2.231 |  | 0.027 |

**Supplementary Fig. 1:** Schematic representation of median developmental stages of common toad tadpoles at the sampling occasions (drawn by Viktória Verebélyi).


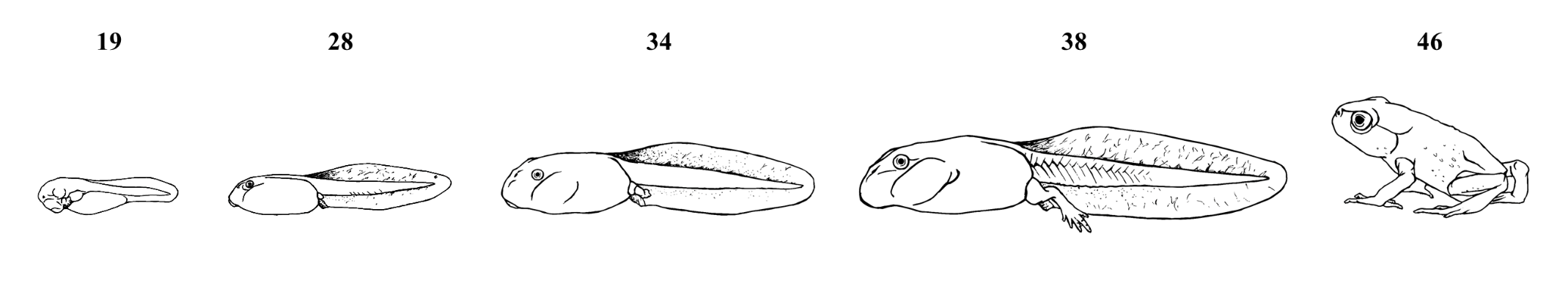


**Supplementary Fig. 2:** Wet body mass of common toad tadpoles during ontogeny in various experimental treatment groups. Mean ± SE are presented (*N* = 303). Asterisks above error bars represent results of pairwise comparisons; groups marked with * (*P* < 0.05) and *** (*P* < 0.001) differed significantly based on linear contrasts corrected for false discovery rate ("†" marks a marginally non-significant difference, *P* = 0.067).


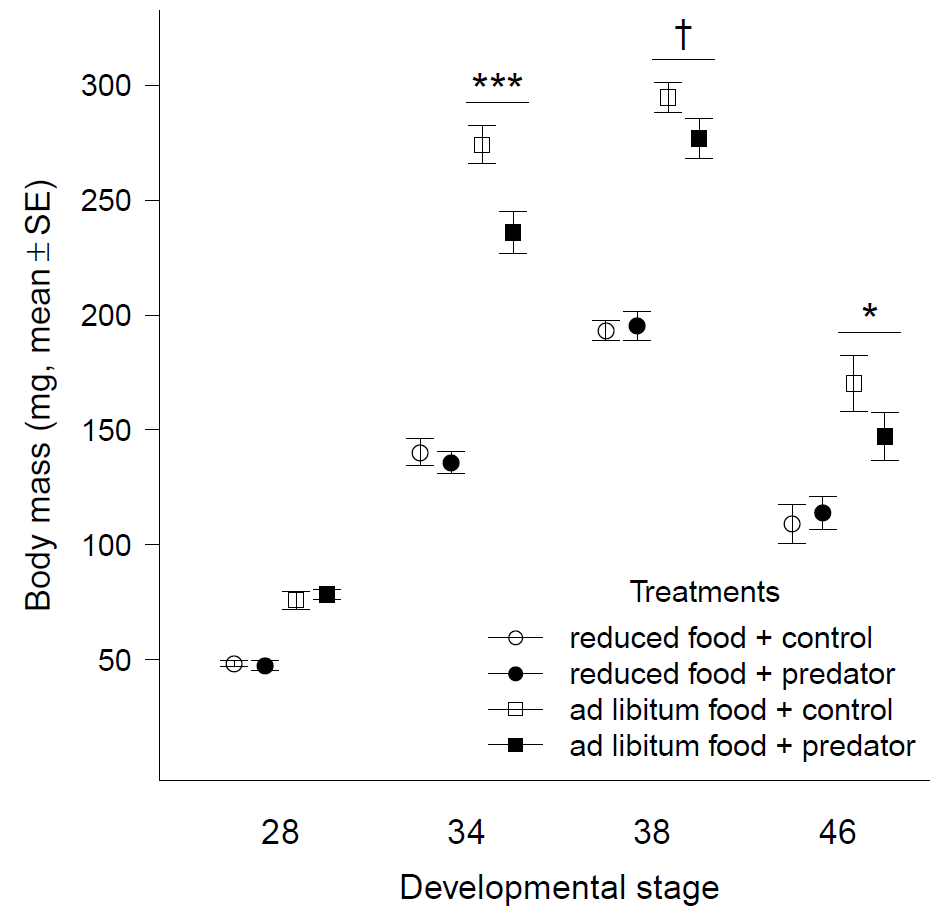


**Supplementary Fig. 3:** Number of bufadienolide compounds of common toad tadpoles by developmental stage and experimental treatments (*N* = 159). In each boxplot, the thick horizontal line and the box represent the median and the interquartile range, respectively; whiskers extend to the upper and lower quartile ± 1.5 × interquartile range; open circles represent outliers. Note the lack of predator effects.

**Supplementary Fig. 4:** Total bufadienolide quantity of common toad tadpoles by developmental stage and experimental treatments (*N* = 158). Note the lack of predator effects.
